# Pediatric hereditary angioedema due to C1-inhibitor deficiency

**DOI:** 10.1186/1710-1492-6-18

**Published:** 2010-07-28

**Authors:** Henriette Farkas

**Affiliations:** 1 3rd Department of Internal Medicine, Faculty of Medicine, Semmelweis University, H-1125 Budapest, Kútvölgyi út 4, Hungary

## Abstract

Hereditary angioedema (HAE) resulting from the deficiency of the C1 inhibitor (C1-INH) is a rare, life-threatening disorder. It is characterized by attacks of angioedema involving the skin and/or the mucosa of the upper airways, as well as the intestinal mucosa. In approximately 50 per cent of cases, clinical manifestations may appear during childhood. The complex management of HAE in pediatric patients is in many respects different from the management of adults. Establishing the diagnosis early, preferably before the onset of clinical symptoms, is essential in cases with a positive family history. Complement studies usually afford accurate diagnosis, whereas molecular genetics tests may prove helpful in uncertain cases. Appropriate therapy, supported by counselling, suitable modification of lifestyle, and avoidance of triggering factors (which primarily include mechanical trauma, mental stress and airway infections in children) may spare the patient unnecessary surgery and may prevent mortality. Prompt control of edematous attacks, short-term prophylaxis and intermittent therapy are recommended as the primary means for the management of pediatric cases. Medicinal products currently used for the treatment of children with hereditary angioedema include antifibrinolytics, attenuated androgens, and C1-INH replacement therapy. Current guidelines favour antifibrinolytics for long-term prophylaxis because of their favorable safety profile but efficacy may be lacking. Attenuated androgens administered in the lowest effective dose are another option. C1-INH replacement therapy is also an effective and safe agent for children. Regular monitoring and follow-up of patients are necessary.

## 1. Introduction

The deficiency of the C1 inhibitor (C1-INH) is inherited as an autosomal dominant trait. It causes hereditary angioedema (HAE-C1-INH), which is regarded as an uncommon disorder characterized by recurrent angioedematous episodes involving the subcutis and/or the mucosa of the upper airways and the gastrointestinal tract [1]. Uncontrolled activation of enzymes belonging to various plasma cascades (such as the complement, fibrinolytic, coagulation, and kinin systems) leads to the release of bradykinin, which contributes angioedema formation by enhancing capillary permeability [2]. The diagnosis of HAE-C1-INH is established by its clinical manifestations, the family history, as well as the findings of complement and molecular genetics studies. Its management consists of the prevention of edematous episodes, as well as the control of acute attacks [3-5]. The range of medicinal products used for prophylaxis (antifibrinolytics, attenuated androgens, and C1-INH concentrate) has not changed for decades. The prophylactic use of plasma-derived C1-INH (pdC1-INH), however, has increased owing to wider availability and other options for emergency intervention have also increased. A kallikrein inhibitor (ecallantide) and a bradykinin B2 receptor antagonist (icatibant) have been introduced to clinical practice and recombinant C1-INH product is under investigation [6,7]. Although the complex management of HAE-C1-INH is in many respects different in children compared to adults, the principles of pediatric therapy are poorly supported by published data with the majority of publications being case reports. The following discussion provides a literature review focused on the hallmarks of pediatric HAE-C1-INH, illustrated by the experience accumulated by the Hungarian HAE Center during the follow-up of 49 children with Type I or Type II HAE-C1-INH (23 males and 26 females with a median age of 6 [4-11] years at diagnosis) from diagnosis to the age of 18 years.

## 2. Diagnosis

In 50 per cent of HAE-C1-INH patients, the manifestations of HAE-C1-INH first occur during childhood. Therefore, establishing the diagnosis early and initiating follow-up care as soon as possible are indispensable for preserving the patients quality of life. The occurrence of edematous manifestations in other members of the patient's family may assist diagnosis. This clue is present in 75 to 85 per cent of cases, whereas in the remaining 15 to 25 per cent, HAE-C1-INH results from a new gene mutation [1]. Within our study population of 49 pediatric patients from 31 families, HAE-C1-INH was diagnosed in first-degree relatives of 41 children (84%) and a new mutation was diagnosed in 8 subjects (16%). According to the Mendelian rules of autosomal inheritance, the offspring of a HAE-C1-INH patient have a 50-per-cent chance of inheriting the disease. Therefore, it is important to establish the diagnosis as early as possible before the onset of clinical manifestations.

### 2.1. Prenatal diagnostics

Prenatal diagnostics may recognize fetal abnormalities requiring intervention *in utero *or during the neonatal period, along with those justifying the termination of pregnancy. Additionally, this diagnostic modality enables parents at genetic risk to avoid passing heritable diseases to their offspring or negative findings may encourage them to have unaffected children. Notwithstanding this, the routine use of prenatal diagnostics in HAE-C1-INH patients is impractical for several reasons. No mutation of the C1-INH gene can be detected in 8 to 10 per cent of cases [8,9]. Identical mutations may be associated with substantially different phenotypes. Additionally, mutation of the C1-INH gene itself may not be a valid indication for terminating pregnancy because it may cause a non-fatal, manageable disease in the offspring, the severity of which may not be predicted in advance. Therefore, abortion should be decided by the mother afflicted by HAE-C1-INH following the evaluation of benefits and risks. Naturally, prenatal diagnostics involving chorionic villous sampling (on weeks 10 to 12) or amniocentesis (on weeks 16 to 18 of gestation) is warranted in the presence of additional risk factors (such as advanced maternal age, AFP abnormality, ultrasound findings suggestive of fetal malformation, atypical number of chromosomes).

Every patient registered with the Hungarian HAE Center undergoes testing of the C1-INH gene at the molecular genetics laboratory of the institution. The data thus obtained are collected in an international, locus-specific database [9]. The Center has established a multidisciplinary team (consisting of an ultrasound expert, a gynecologist, and a geneticist) for implementing prenatal diagnostics. Remarkably, only a single patient has contemplated this option but refrained from using it eventually. The changes of attack frequency during the last trimester of pregnancy are of potential prognostic value. According to our observations, pregnancy with a fetus affected by HAE-C1-INH was associated with a significant (p = 0.039) increase in the number of edematous attacks experienced by the mother during the third trimester [10].

### 2.2. Postnatal diagnosis

#### 2.2.1. Symptom-free children with a positive family history: initial screening (including complement tests) is necessary at the age of 6 months and one year

Type I HAE-C1-INH is characterized by reduced C4, as well as reduced antigenic and functional C1-INH levels. In Type II HAE-C1-INH C4 is reduced and antigenic C1-INH level is high or normal and the functional activity of C1-INH is reduced [4,5]. Complement concentrations measured in cord blood from full-term neonates are lower than maternal levels. Antigenic and functional C1 inhibitor levels correspond to 70% and 61.8% of adult values, respectively [11] and increase to the normal level by the age of 6 months to one year. Therefore, too early testing may lead to a false diagnosis of HAE-C1-INH. Furthermore, serum complement levels are influenced by birth weight and gestational age [12,13]. In conformity with international recommendations, the screening of pediatric patients with a positive family history is performed at the Hungarian HAE Center after the age of 6 months and repeated after the patient has turned one year [5]. Half of our patients (26 out of 49) were symptom-free at the time of diagnosis with screening prompted by positive family history for angioedema (Table 1).

**Table 1:** Demographical data of and the circumstances of establishing the diagnosis in the study population.

	N° of patients		Age (median) at diagnosis	Age at the onset of symptoms
			
	Boys	Girls		
**Symptom-free, identified by family screening (53%)**	13	13	5 (3-11)	6 (4-12)

**Symptomatic, identified by family screening undertaken after either parent had been diagnosed with HAE-C1-INH (26%)**	7	6	7 (5-12)	5 (3-10)

**Diagnosis established by clinical manifestations (21%)**	3	7	9 (4-11)	3 (1-7)

25^th ^and 75^th ^percentiles are shown in parentheses.

Although HAE-C1-INH results from mutation of the C1-INH gene, molecular genetic analysis is not a prerequisite for diagnosis, as a complement study is sufficient to recognize the disease [5]. In addition to its fundamental role in prenatal diagnostics, molecular genetics analysis may aid the early diagnosis of uncertain cases at the age of 1 to 3 years. In our practice, this method has proven extremely helpful in two children whose C4 levels were normal, whereas C1-INH antigenic concentration and functional activity were borderline low at initial and follow-up measurements. DNA analysis detected the mutation identified earlier in the parental C1-INH gene in both children and thus established the diagnosis. Subsequently, the characteristic symptoms of HAE-C1-INH manifested in both children.

#### 2.2.2. HAE-C1-INH suggested by the symptoms of the child

The diagnosis of HAE-C1-INH was suggested by clinical manifestations in 21 per cent (10/49) of our pediatric patients and in five of these, a negative family history interfered with the recognition of the hereditary disorder (Table 1). Following the identification of affected parents, family screening led to diagnosing HAE-C1-INH in 26 per cent (13/49) of children exhibiting edematous symptoms (previously attributed to allergy). In 9.2 per cent (4/49) of cases, establishing the diagnosis of HAE-C1-INH in the offspring shed light on the obscure etiology of edematous episodes experienced by either parent or identified the latter as an asymptomatic carrier. The 'lag period' between the onset of symptoms and the diagnosis of HAE-C1-INH (an efficiency marker of the health care delivery system managing the patients) is highly variable among different countries. The mean duration of this lag period until diagnosis is 21 years according to FRANK [14] and 16.3 years according to BYGUM [15]. By contrast, it was 11.2 years in our study population and very short - just 2.36 years - in the subset of symptomatic children. The latter is explained by the fact that the complete range of complement tests are performed at the HAE-C1-INH Center on every patient with angioedema of unknown etiology.

##### Clinical manifestations

Although angioedematous episodes may occur at any age, these usually begin between 5 and 11 years. Mean age at disease onset was found to be 11.2 years by BORK [16], 9.5 years by, BYGUM[15], and 4.4 years by MARTINEZ [17], whereas it was 6.6 years in our patient population. Clinical symptoms are extremely uncommon during infancy [11]. Edema may involve the subcutis or/and the submucosa. Subcutaneous edema appears on the extremities, the face, neck, torso and genitals as a non-pruritic and non-erythematous lesion. It is the most common and the earliest localization in pediatric patients: it was the initial manifestation of HAE-C1-INH in 27 of our 49 patients. Subcutaneous angioedema usually resolves spontaneously within 2 to 4 days [16].

When angioedema involves the larynx, submucosal edema of the upper airways can lead to asphyxia. The visual appearance of edema is not different from that seen in upper airway edema (UAE) of other inflammatory or allergic etiologies. According to case reports published in the literature, it was misdiagnosed as edema caused by allergic asthma in a three-year-old girl [18] and mistaken for epiglottitis in another child [19]. However, there is a helpful differential diagnostic clue: standard medications used to relieve airway edema (such as glucocorticoids, antihistamines, and epinephrine), which usually accomplish dramatic improvement in children compared to adults, tend to be ineffective in reducing the swelling related to HAE [20,21]. In comparison to adults, asphyxia may ensue more rapidly in children because of smaller airway diameter. Additionally, laryngoscopy is more difficult to perform in small children owing to the lack of co-operation [22]. Angioedema of the larynx is rare: 0.9 per cent of all HAE-C1-INH attacks. Almost 80 per cent of UAEs occur between the age of 11 and 45 years, although this condition has been described in a child as young as 3 years [23]. In our study population, the earliest time of onset of UAE was similarly 3 years of age. However, it was not an initial manifestation in any of our patients. Up to the age of 18 years, 23 of our 49 patients sustained at least one attack of UAE and the greatest number of upper airway episodes experienced by the same patient was 43. Edema confined to the tongue did not occur in our patients.

In the gastrointestinal tract, submucosal edema may be associated with colicky abdominal pain, nausea, vomiting, watery post-attack diarrhea, occasional paleness of the skin consequent to hypovolemia, prostration, dehydration, tachyarrhythmia, and fainting and it may mimic an 'acute abdominal catastrophe'. Afflicted patients are usually admitted to a surgical department for observation and are often subjected to an unnecessary operation [24]. The most likely differential diagnostic candidates include acute appendicitis, mesenteric lymphadenitis, intussusception, strangulation ileus resulting from intestinal torsion and less often suspected perforated Meckel's diverticulum, polycystic ovarian syndrome with ovarian torsion, hemorrhage or infarction [25]. Edema of the intestinal wall may lead to intussusception [26-28].

Recurrent abdominal complaints of unknown etiology should always raise the suspicion of HAE-C1-INH. The clinical manifestations of an edematous attack of HAE-C1-INH are accompanied by intra-abdominal abnormalities detectable by diagnostic imaging (ultrasound, US; CT; video capsule endoscopy; etc.). Although US findings (including free peritoneal fluid, edema of the intestinal wall, and abnormalities of liver structure) are non-specific to HAE-C1-INH, abdominal US may prove a sensitive, rapid, and non-invasive differential diagnostic modality, which is particularly straightforward in pediatric patients [29,30]. Edematous attacks are not associated with any specific laboratory abnormality although leukocytosis can occur particularly with hemoconcetration. The attack is not accompanied by pyrexia and the laboratory parameters of inflammation are normal. As the edematous attacks of HAE-C1-INH are associated with elevated prothrombin fragment 1+2 (F1.2) and D-dimer levels, these indices may serve as objective, but non-specific biomarkers [31].

An edematous abdominal attack occurred as the initial manifestation of HAE-C1-INH in 3 of the 49 pediatric patients. Estimating the prevalence of intestinal edema in the pediatric population is difficult, because 'belly ache' is a common symptom with a multitude of possible causes, especially in infants. One to two per cent of HAE-C1-INH attacks can occur in other localizations including the urinary bladder, the urethra, muscles and joints, kidney; pericardial or pleural effusion (known as the 'chest episode') or neurological symptoms [1,16]. In our study population (n = 49), a 'chest episode' evolved in three and pericardial effusion occurred in one patient during an attack [32].

The characteristic prodromal sign of erythema marginatum (appearance of a map-like pattern on the skin) occurs more frequently during childhood. In a proportion of cases, this lesion can evolve as an independent phenomenon, without a subsequent attack. In our study population, this symptom occurred in 42 per cent of patients [33], whereas BYGUM observed it in 58 per cent of cases [15]. Its appearance is a potential differential diagnostic pitfall, because a similar skin lesion can evolve in viral or bacterial infections, as well as it can be misdiagnosed as urticaria [34].

The time of onset, frequency, and duration of symptoms, as well as the severity of attacks all exhibit inter-individual variation and substantial differences exist even within the same family. Analyzing the time of onset of disease symptoms revealed an increase in the frequency and severity of manifestations between 3 and 6 years of age, as well as around puberty. This is probably related to the manifold physiological (endocrine, mental, and somatic) changes occurring in these periods of development. In agreement with the observations of BORK, we found that the earlier the onset of symptoms, the more severe will be the subsequent course of HAE-C1-INH [16].

## 3. Management

### Counselling

Counselling the patient and family is the initial step after diagnosis. Providing the patient and family with appropriate information is indispensable to adopting a suitable lifestyle and avoiding severe complications. At the initial visit, the child and parents are counselled in person and ongoing consultation is offered using a telephone hotline and the website of the HAE Center http://www.haenet.hu.The kindergarten or school are informed of the diagnosis in writing. The patient receives a multilingual information card to carry at all times. Additionally, special medication for emergency use at home during acute edematous attacks is provided, along with contact information on the self-help organizations of patients.

### 3.1. Primary prevention

By triggering edematous attacks, certain factors can influence the time of onset and localization of symptoms. Provoking factors include trauma, emotional stress, surgery or diagnostic manipulation of the head and neck region, physiological changes of sexual hormones (during puberty, the menstrual cycle or pregnancy), changes of the weather, specific foodstuffs and medicinal products [1]. The incidence of these factors differs slightly among pediatric and adult patients. The initial phase of therapy is primary prevention - that is, the identification and when possible elimination of triggering factors. The exploration of the latter in our study population identified mechanical trauma as the most common provoking factor (52.6%), followed by mental stress (36.8%), airway infection (36.8%), and menses (26.7%). By contrast, physical exertion (60.4%), mental stress (56.6%), mechanical trauma (53.8%), pregnancy (39.4%), and menses (26.8%) are the most common triggering factors in adults. A proportion of attacks can be prevented through appropriate counselling and changes to lifestyle [10]. This is supported by our observation that in patients whose disease had been diagnosed before the onset of symptoms, initial manifestations occurred later, at the age of 6 (rather than 4) years. We recommend that children with HAE-C1-INH participate in sports regularly, but activities involving direct bodily impact are not recommended. Therefore, only partial exemption from school gymnastics is advised. Considering that infection is an important triggering factor in the pediatric population, infants should not attend peer communities (e.g. nursery school, kindergarten) during the period of age-related susceptibility to infections. *Helicobacter pylori *is another potential provoking factor. Accordingly, it is expedient to screen patients for infection by this bacterium and when necessary, administer eradication therapy even during childhood [35].

If despite counselling, the manifestations of HAE-C1-INH recur with increasing frequency and in more severe form, it is important to search for other accompanying disorders. In children, abdominal symptoms and/or neurological signs may suggest celiac disease. Certain medicinal products (such as estrogen-containing oral contraceptives, ACE inhibitors, ARBs) can also induce the manifestations of HAE-C1-INH. Estrogen containing contraceptives are not recommended for adolescent girls. ACEIs and ARBs are widely administered to adult patients, but data on their pediatric use are limited. Only two case reports have been published on ACEI-induced angioedema in children [36,37] and no information is available from the literature on pediatric patients with HAE-C1-INH. Immunizations are usually recommended for children with HAE-C1-INH and the prevention of infections may reduce the frequency of edematous attacks.

### 3.2. Drug prophylaxis

As disease manifestation onset usually occurs between 6 to 8 years of age, prophylaxis is extremely uncommon under the age of 6 years [1]. During this period of life, emergency therapy of acute attacks is preferred and pharmacologic intervention should be initiated as early as possible at the onset of the attack. Other alternative form of short-term prophylaxis may then be considered. As regards the pediatric population of HAE-C1-INH patients diagnosed by our team, 61.23% (30/49) did not require therapy after diagnosis, owing to the lack of symptoms.

#### 3.2.1. Short-term prophylaxis

This prophylactic modality involving treatment on a single occasion or over several days is intended for the prevention of a single impending attack.

##### 3.2.1.1. 'Classical' short-term prophylaxis

This type of prophylaxis is recommended before surgical, diagnostic interventions contemplated in the head and neck region, including dental procedures, and other operations performed under endotracheal manipulation, as these may trigger an edematous attack -UAE primarily [4,5]. The following agents are appropriate: danazol 5 mg/kg/day (maximum daily dose is 600 mg); tranexamic acid 20 to 40 mg/kg/day in 2 or 3 divided doses (maximum daily dose is 3 g) introduced 5 days before and continued for additional 2 days after the intervention; and C1-INH concentrate 10 to 20 U/kg administered one hour before the procedure [5,38]. Fresh frozen or solvent-detergent plasma may be used only if C1-INH concentrate is not available [39] and in poor-risk patients undergoing major surgery. The recommendations by GOMPELS *et al *on the duration of prophylaxis and of C1-INH supplementation are at variance with our practice [4].

During childhood, surgical interventions are less frequent, shorter in duration and may not necessarily require general anesthesia. Operations performed under intratracheal narcosis were identified in the history of eleven patients - eight of these had experienced an edematous attack after the intervention before their HAE-C1-INH was recognized. After diagnosis, short-term prophylaxis was administered before 5 procedures (ENT intervention, dental extraction, appendectomy under ITN) performed in 4 patients. Patients on pre-existing treatment with tranexamic acid or danazol received these agents in escalated doses before minor procedures. Untreated patients received 500 U C1-INH concentrate one hour before major operations and another 500 U was kept ready during all such interventions. Short-term prophylaxis (as above) invariably prevented edema formation; the postoperative period was uneventful and no complications ensued [10]. Since its marketing authorization in Hungary, only C1-INH concentrate is administered at the HAE-C1-INH Center before major surgery or diagnostic procedures.

##### 3.2.1.2. 'Alternative' short-term prophylaxis

This type of prophylaxis is used in the presence of prodromal symptoms as well as when potentially edema-inducing pathological, physiological or environmental effects persist for a brief (several-hours/days-long) period only. Upper respiratory track infections occur frequently during childhood and it is important to treat bacterial infections early and to introduce short-term prophylaxis as soon as possible and to continue its administration over the duration of the infection [38]. In our practice, this type of prophylaxis was occasionally started at an appropriate time during the menstrual cycle and continued for a week - or optionally, a single dose of C1-INH concentrate was administered. The choice between these options depended on the particular phase of the menstrual cycle, identified as critical (see the agents and their dosages in the section on 'classical' short-term prophylaxis).

When prodromal symptoms (pruritus, tingling, nausea, dry mouth, heartburn, diarrhea, anxiety, fatigue, erythema marginatum, joint-pressure sensation) occurred, tranexamic acid (40 mg/kg/day) or danazol (100-200 mg/day) administered over 2 to 3 days reduced the severity and halved the duration of subcutaneous or gastrointestinal manifestations [38].

#### 3.2.2. Long-term prophylaxis (LTP)

The literature reveals that the recommendations on introducing long-term prophylaxis are extremely heterogeneous [4,5,14,40-44]. In general, severe or frequently recurring attacks are considered among the indications for long-term prophylaxis. UAE in the patient's history was included as a criterion by 4 out of 7 authors [4,5,40,42]. The methods for determining attack frequency were variable. Daily activities were taken into account by 4/7 authors [14,40,42,43]. The UK guideline added the requirement of "concentrate administration" as an additional criterion (although "acute treatment" would have been more appropriate). Limited access to medical care was mentioned in two proposals [40,42].

Drugs suitable for long-term prophylaxis include antifibrinolytics (epsilon-aminocaproic acid, tranexamic acid), attenuated androgens (danazol, stanozolol, oxandrolone) and C1-INH concentrate [4,5]. Experience with long-term C1-INH prophylaxis is limited in pediatric patients. In 2009, a new, plasma-derived C1-INH concentrate was approved in the USA for long-term prophylaxis. The expanding range and indications for the use of C1-INH preparations along with their increasing availability are expected to encourage the use of - as well as obtaining further clinical experience with - these products [45,46]. Currently, consensus statements by various authorities unanimously advocate tranexamic acid (TXA) as the agent of choice for long-term prophylaxis in children because its safety profile is more favorable than that of attenuated androgens [38,47]. Attenuated androgens may be administered when antifibrinolytics are ineffective or contraindicated (i.e. for patients with a history of thromboembolism or a family history of thrombophilia) [38]. Adverse reactions can be avoided by administering the lowest effective dose [48-52].

Androgen side effects include decreased growth rate, virilization, and behavioral disorders during childhood, whereas in adolescence, menstruation irregularities and elevation of serum transaminase levels may occur [53]. Weight gain, myalgia, headache, libido changes, microhaematuria, alterations of lipid profile, and the development of hepatocellular carcinoma or adenoma are more characteristic of adult patients, as the safety profile of danazol is related to its dose and the duration of its use [54].

The number of longitudinal studies into the effectiveness and adverse effects of LTP is limited in both adult and pediatric patients. LTP is introduced if UAE has been detected in the history, as well as the patient has experienced severe and recurrent attacks with no identifiable triggering factor. After diagnosis, the frequency and severity of disease manifestations warranted LTP in 18.36% (9/49) of our pediatric patients - in contrast to the management of adults, where 39% are receiving LTP. TXA 20 to 40 mg/kg daily was administered in two or three divided doses (maximum dose: 3 g/day split 2 or 3 times per day). Before TXA was available, epsilon-aminocaproic acid (EACA, 0.17-0.43 g/kg per day) had been used, but it was less well tolerated by patients than TXA, which is now preferred for initial prophylaxis.

When antifibrinolytics failed to achieve satisfactory improvement, caused severe adverse effects, or their use was contraindicated, treatment with attenuated androgens was introduced according to the Budapest protocol [5], with titration to the lowest effective dose level. Treatment with the lowest effective maintenance dose of danazol (2.5 mg/kg per day; 50 mg/day initial dose) was started and if effective reducing the dosage interval to every other day or every third day. If necessary, the dose was increased to a maximum of 200 mg/day. Potential adverse effects were monitored.

Danazol was well tolerated by our pediatric patients [55]. Treatment with this drug was effective; it reduced the number of attacks significantly during the first year. Unfortunately, however, the effect of danazol declined during the 4^th ^and 5^th ^years of therapy despite the escalation of its dose and this is similar to our experience with the treatment of adult patients [10] . Notwithstanding this, the majority of patients treated with these agents are not completely symptom-free. All patients treated with C1-INH supplementation received prophylaxis with antifibrinolytics or attenuated androgens.

#### 3.2.3. 'Intermittent' prophylaxis

Long-term prophylaxis does not necessarily mean uninterrupted dosage with the drug over a lifetime - although this issue is not enlarged upon in consensus guidelines. On occasion of annually scheduled, regular check-ups, the therapeutic regimen is modified frequently: drugs are discontinued and others are introduced, as well as their doses are adjusted. Although it is not mentioned by pertinent guidelines, intermittent prophylaxis may prove effective and safe, especially in the management of pediatric patients. The technical term "intermittent prophylaxis" was used in relation to danazol treatment by AGOSTONI as early as in 1978 already [56]. We used this modality of prophylaxis when a change occurred in the number or severity of edematous attacks and although the underlying causes of this change were suspected, their elimination was not possible. Additionally, 'intermittent' prophylaxis was administered during prolonged, critical periods known to be associated with attacks (such as starting school, exam periods, outbreaks of upper airway infections, winter months, family problems, adolescence, pregnancy). Occasionally, the drugs conventionally used for LTP were administered in combination with intermittent C1-INH substitution (1 to 2 × 500 U/week). Intermittent prophylaxis with C1-INH was introduced when discontinuation of danazol had become necessary owing to lack of effect or the occurrence of undesirable effects. Intermittent prophylaxis with TXA and C1-INH concentrate administered to 20.41% (11/49) of our patients prevented edematous attacks - or at least reduced their duration and severity substantially.

#### 3.3. Emergency therapy

The management of full-blown attacks depends on their localization and severity. The range of drugs suitable for emergency use has increased over recent years. In addition to the medicinal products used earlier (antifibrinolytics, attenuated androgens, C1-INH concentrate, fresh frozen or solvent-detergent plasma), icatibant (a bradykinin receptor B2 antagonist) and ecallantide (a kallikrein inhibitor) have become available in clinical practice and recombinant C1-INH is under clinical trial. No experience is available yet with the pediatric use of the latter three innovative drugs, which differ from previous treatments in dosage, mode of action, and manufacturing process. By increasing the range of therapeutic alternatives, the introduction of these agents enables medical professionals to make much more specific and individualized decisions in choosing an appropriate treatment [7]. Owing to their straightforward administration by subcutaneous injection and prompt effect, both the kallikrein inhibitor and the bradykinin receptor antagonist are expected to prove extremely beneficial for pediatric patients. In our expectations, treatment with these agents might obviate the need for introducing long-term pharmacotherapy and can relieve patients from taking oral medication continuously and occasionally over a lifetime.

Although usually resolving spontaneously, subcutaneous attacks can be controlled by treatment with antifibrinolytics and anabolic steroids. Administering increased doses of these drugs reduces the duration of attacks and prevents their escalation. Edema localized to the extremities does not progress to a severe condition but in pediatric patients it is a common cause of absenteeism from school (and adults from work). Edematous swelling of the face, lips, neck or torso, as well as substantial edema of the extremities require special management. Facial edema may progress and involve the mucosa of the upper airways. Edema of the neck can cause complications through compression, whereas subcutaneous swelling on the chest may be accompanied by pericardial or pleural effusion [16,32,57]. Severe edema of the extremities is very painful and can interfere with blood circulation in the affected limb. Following published guidelines, we always administered C1-INH concentrate, which mitigated symptoms within 30-60 minutes and eliminated them completely over 24 to 48 hours. Treatment with C1-INH concentrate was necessary for edema of the extremities in two pediatric patients [58].

Edema of the upper airway mucosa is a life-threatening condition potentially leading to asphyxia, which is responsible for the 30-to 40-per-cent mortality related to lack of treatment or delayed diagnosis [59]. Currently, the administration of C1-INH concentrate is the only adequate treatment for children with UAE [4,5,38]. In our practice, edema of the upper airway mucosa is similarly relieved by administering C1-INH immediately. The child is then hospitalized until the complete resolution of symptoms, in an institution where an ICU is available with ready access to endotracheal intubation (or tracheostomy if needed) and airway management. The initial dose of C1-INH concentrate is 10 to 20 units/kg for pediatric patients (usually 500 units). Symptoms subsided markedly within 15 to 30 minutes and resolved completely within 12 hours after treatment. No recurrence of the attack nor occurrence of undesirable effects were observed. Earlier administration of C1-INH during an attack was followed by more rapid regression. Accordingly, the education of patients was particularly focused on the accurate description of subjective symptoms of UAE, stressing that mild dysphagia and globus sensation are the most common initial manifestations [58].

Edema of the gastrointestinal mucosa requires emergency treatment to relieve acute clinical symptoms (intense and colicky abdominal pain, nausea and vomiting) and to correct hypovolemia from post-attack watery diarrhea. Additionally, clinical manifestations may mimic the signs of an 'acute abdominal catastrophe', which warrants considering the need for surgical intervention. In patients with known HAE-C1-INH, the dramatic effect of C1-INH concentrate administered for abdominal symptoms may be of differential diagnostic value. In our experience, treatment with this agent is followed by substantial mitigation of symptoms within half an hour and their complete resolution within 12 to 24 hours. The abnormalities detected by abdominal US (such as free peritoneal fluid, edema of the intestinal wall, 'starry sky' pattern in the liver) also resolve within 24 to 48 hours [29,30]. The initial dose of 500 U was sufficient even in abdominal attacks and repeating this dose within 4 hours owing to unsatisfactory improvement was necessary in only two children. The maximum dose should not exceed 20 U/kg. C1-INH is appropriate for any population and all age groups of pediatric patients. In view of the increased susceptibility of children to hypovolemia and considering the substantial extravasation into the peritoneal cavity and the intestinal lumen, we always administer physiological saline as parenteral fluid replacement during every abdominal attack. Treatment with C1-INH concentrate has been effective, not accompanied by adverse effects or the transmission of infection, nor antibody formation. We no longer administer fresh frozen plasma since C1-INH concentrate has become available 20 years ago [38,58].

#### 3.4 Home-based management

This involves prophylactic or emergency treatment in the patient's home, administered by the summoned health care professional or as self-medication by the patient or family member. This modality has the advantage of the fastest possible treatment without the delay incurred by transportation of the patient to a health care institution. Naturally, a prerequisite to this approach is to ensure the constant availability of emergency medication in the patient's home [60-62]. No consensus has been reached yet regarding home-based management. At the 6^th ^C1-INH Deficiency Workshop (held between 22 and 24 May 2009 in Budapest, Hungary) a roundtable conference discussed the topic of self-injection by patients, in order to lay the foundations of future international guidelines.

As regards pediatric patients, medication is best administered by a health care professional. Expert assessment of the patient's condition, observation of symptomatic improvement, and intravenous drug administration are best handled by experienced professionals. In case of UAE, emergency treatment at home should be followed by hospitalization until symptoms resolve completely. Admission to hospital is similarly necessary in a severe abdominal attack, in order to rule out a possible, acute abdominal emergency. If a life-threatening condition has occurred and no expert help is available within reasonable time, the emergency medication may be administered by parents, relatives, or older pediatric patients themselves - on condition that they have acquired the technique of intravenous injection beforehand [38]. The success of home-based management of 12 pediatric patients was reported by MARTINEZ [17]. In Hungary, C1-INH concentrate was approved in 1996. The Ministry of Health has made this medication available free of charge to all HAE-C1-INH patients. Patients are allowed to keep the concentrate at home and therefore, it is constantly available at hand for administration by the general practitioner on duty or the summoned emergency medical technician. In Hungary, family practitioners are authorized to prescribe C1-INH concentrate on proposal from the principal of the HAE center.

#### 3.5. Follow-up

As HAE is an hereditary disorder and its gene therapy is not yet available, patients must reconcile themselves to a lifelong disease experience and prolonged doctor-patient relationships. The delivery of follow-up care for HAE-C1-INH patients and the accumulation of data on their disease and management are best implemented using a centralized approach [38]. HAE centers should be established in consideration of the conditions prevailing in the country involved. In Hungary, for example, a single center is sufficient owing to the size of the population and geographic properties of the country. The National HAE Center consists of the following five organizational units: outpatient clinic, inpatient facility for the emergency therapy of adult and pediatric patients, complement laboratory, molecular genetics laboratory, and the self-help organization of HAE-C1-INH patients [63]. In addition to diagnosing HAE-C1-INH accurately, counselling and educating patients, as well as choosing and prescribing the medication most appropriate for individual patients, the Center must also assume the follow-up care of patients. The latter involves a control visit at least once a year; however, newly diagnosed patients and those on LTP should be monitored at 3-month intervals initially and then, twice a year for the next 2 years. Control visits comprise a laboratory screen, anthropometric assessment, and abdominal US, as well as the recording of symptoms and potential undesirable effects associated with drug treatment (by reviewing patient diaries and hospital discharge summaries) - all these afford adjusting therapy and introducing new treatments as necessary. At the Hungarian HAE Center, we use mail notification to summon patients for control visits (minors are to be accompanied by their parents). Compliance is excellent: a mere 2% of our 132 followed-up patients do not return for control visits. Between visits, professional support is available to patients via telephone or e-mail and uninterrupted exchange of information is maintained with the family practitioner or pediatrician of the patient.

The diagnosis, management, and follow up of pediatric patients with HAE-C1-INH are different from those of adults. Familiarity with specific, childhood properties is indispensable to making an accurate diagnosis, choosing the most appropriate therapy, and shaping the pediatric patients' lifestyle to enable them to live a fuller life similar to their healthy peers.

## Competing interests

The author declares that they have no competing interests.
